# Supplementary material for: The MOM1 complex recruits the RdDM machinery via MORC6 to establish de novo DNA methylation
Source: Nat Commun. 2023 Jul 12;14:4135. doi: 10.1038/s41467-023-39751-4 (PMC10338684; doi:10.1038/s41467-023-39751-4)
Supplement: Supplementary file 1 — Supplementary Information [file 41467_2023_39751_MOESM1_ESM.pdf]

The MOM1 complex recruits the RdDM machinery *via* MORC6 to establish *de novo* DNA methylation

Li *et al.*

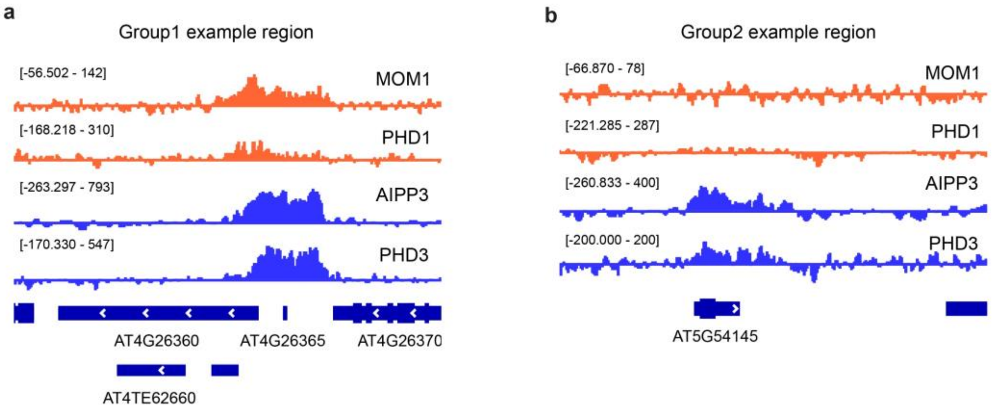

Supplementary Figure 1. Example of AIPP3 Group1 and Group2 ChIP-seq peaks. Screenshots of MOM1-Myc, PHD1-FLAG, AIPP3-FLAG and PHD3-FLAG ChIP-seq signals over representative AIPP3 Group1 peaks (**a**) and Group2 peaks (**b**), with control ChIP-seq signals subtracted.

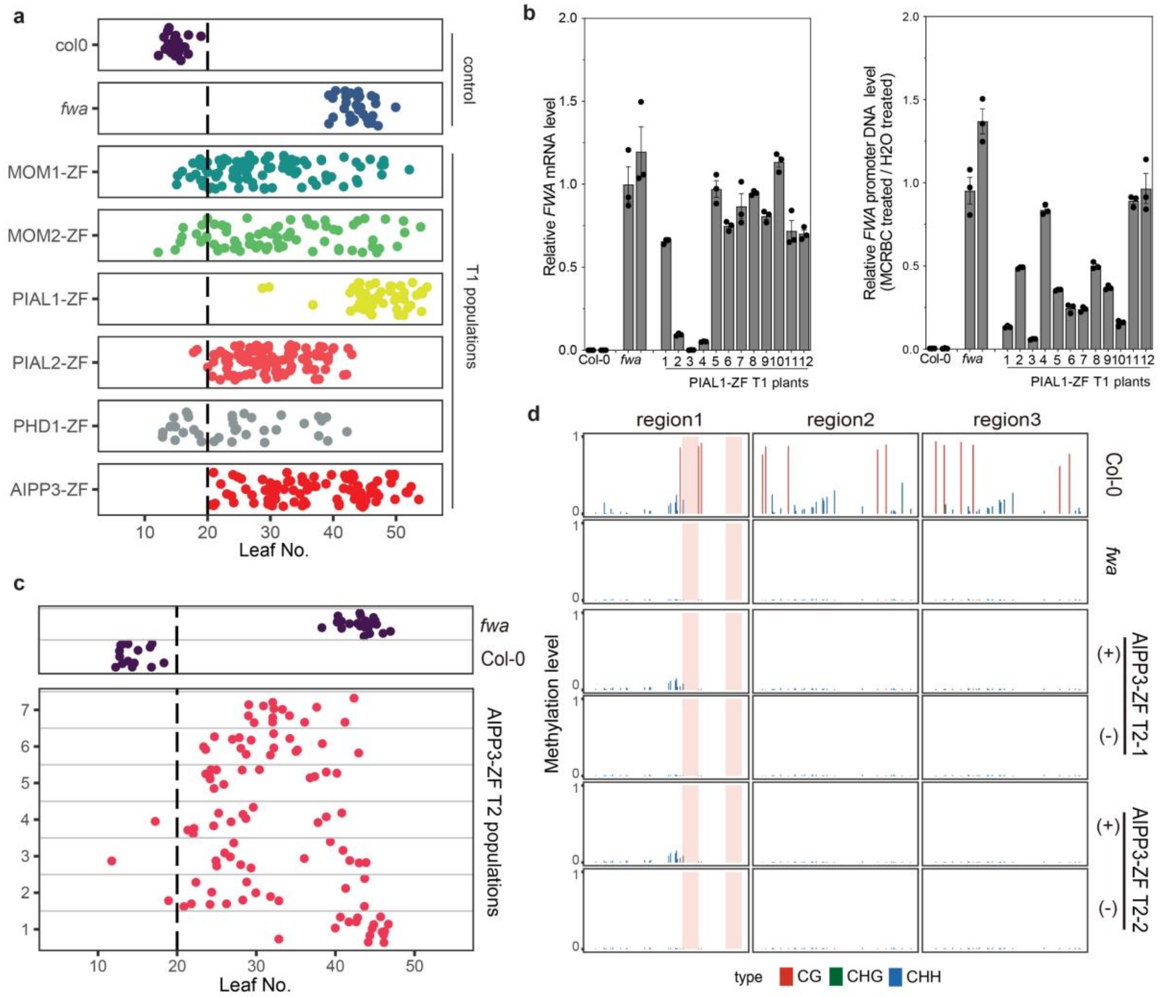

Supplementary Figure 2. PIAL1-ZF and AIPP3-ZF silence *FWA* less efficiently than ZF tethering of other MOM1 complex components. a, Flowering time of Col-0, *fwa*, and T1 populations of MOM1-ZF, MOM2-ZF, PIAL1-ZF, PIAL2-ZF and PHD1-ZF in the *fwa* background. **b**, left panel: qRT-PCR showing the relative mRNA level of *FWA* gene in PIAL1-ZF T1 plants in *fwa* background. Right panel: qPCR showing the relative *FWA* promoter DNA quantity after McrBC treatment in PIAL1-ZF T1 plants in *fwa* background. Bar plots and error bars indicate the mean and standard error of three technical replicates, respectively, with individual technical replicates shown as dots. **c,** Flowering time of *fwa*, Col-0, and representative T2 populations of AIPP3-ZF in *fwa* background. **d**, CG, CHG, and CHH DNA methylation levels over *FWA* promoter regions measured by BS-PCR-seq in Col-0, *fwa* and representative T2 plants of AIPP3-ZF with (+) or without (-) transgenes in the *fwa* background. Pink vertical boxes indicate ZF binding sites. For **a** and **c**, the numbers of independent plants (*n*) scored for each population and detailed statistics of flowering time comparison between different populations are listed in Supplementary Data 5. Source data are provided as a Source Data file.

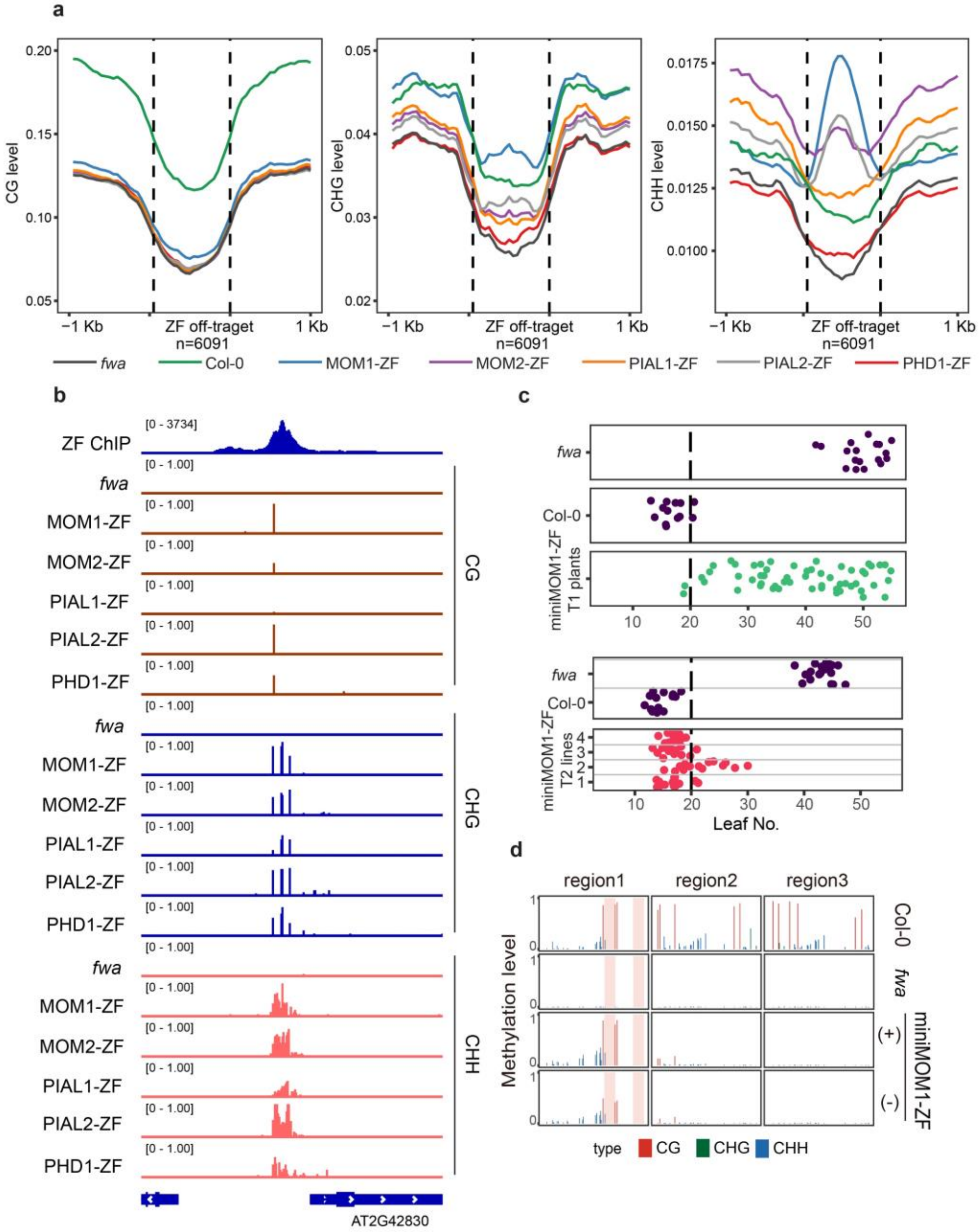

Supplementary Figure 3. ZF tethering of MOM1 complex components and miniMOM1 leads to DNA methylation. a, Metaplots showing DNA methylation levels of CG, CHG, and CHH contexts over ZF off-target sites in representative T2 plants of MOM1-ZF, MOM2-ZF, PIAL1-ZF, PIAL2-ZF and PHD1-ZF in the *fwa* background, the *fwa* control plants and the Col-0 plants (GSM2124018) measured by whole genome bisulfite sequencing (WGBS). **b**, Screenshots of Whole Genome Bisulfite Sequencing (WGBS) showing CG, CHG, and CHH DNA methylation level over a representative ZF off-target site in *fwa*, and representative T2 plants of MOM1-ZF, MOM2-ZF, PIAL1-ZF, PIAL2-ZF and PHD1-ZF in the *fwa* background. **c**, Flowering time of miniMOM1-ZF T1 plants in the *fwa* background (upper panel) and representative T2 lines (lower panel). The numbers of independent plants (*n*) scored for each population and detailed statistics of flowering time comparison between different populations are listed in Supplementary Data 5. **d**, CG, CHG, and CHH DNA methylation levels over *FWA* promoter regions measured by BS-PCR-seq in Col-0, *fwa*, and representative mini-MOM1-ZF T2 plants with (+) or without (-) miniMOM1-ZF transgenes in the *fwa* background. Pink vertical boxes indicated ZF binding sites. Source data are provided as a Source Data file.

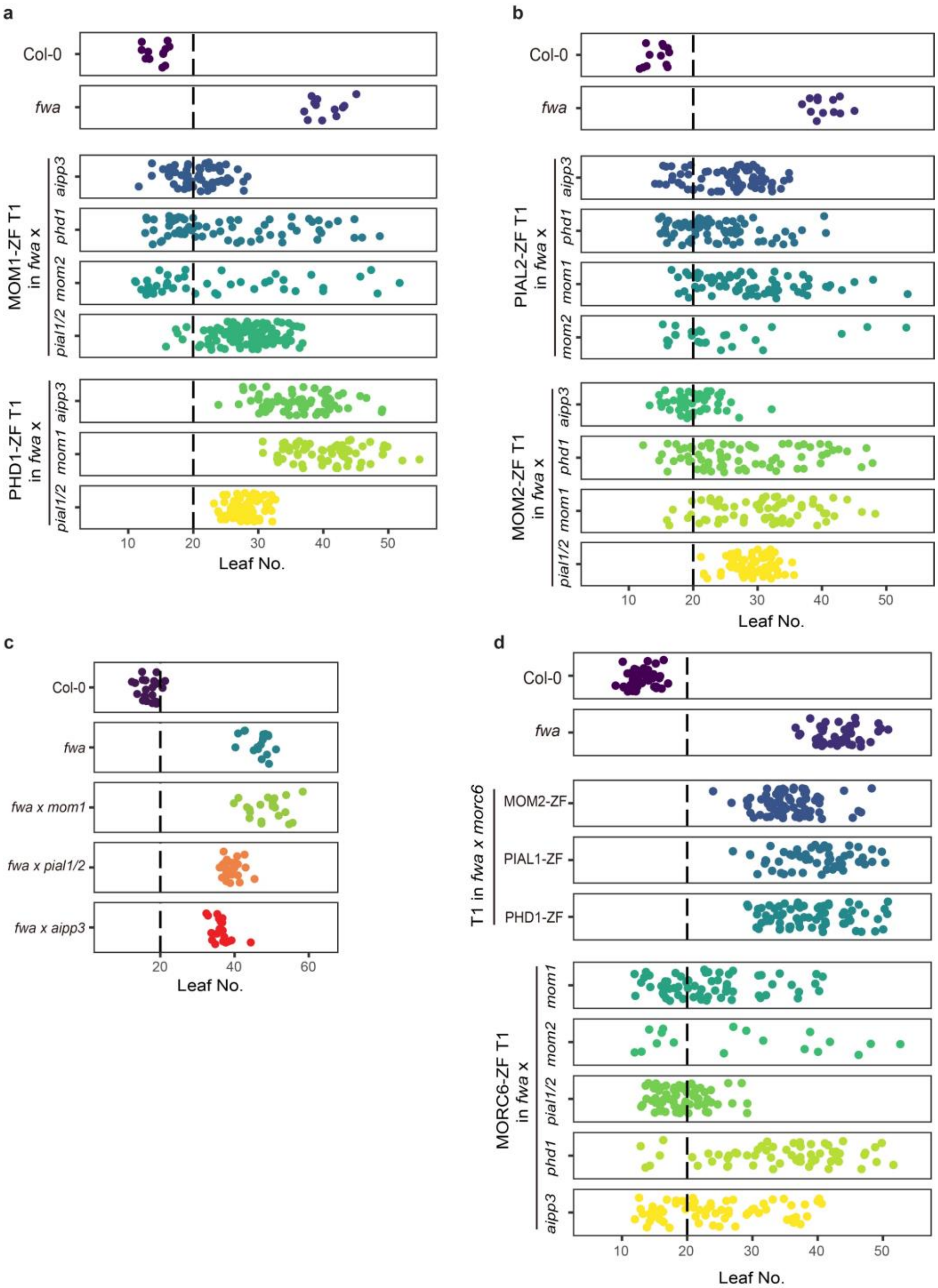

Supplementary Figure 4. Analysis of ZF tethering of MOM1 complex components and MORC6 in mutant backgrounds. **a**, Flowering time of MOM1-ZF T1 plants in the backgrounds of *fwa* introgressed into *aipp3-1*, *phd1-2*, *mom2-1* and *pial1/2* mutants; Flowering time of PHD1-ZF T1 plants in the backgrounds of *fwa* introgressed into *aipp3-1*, *mom1-3* and *pial1/2* mutants. **b,** Flowering time of PIAL2-ZF T1 plants in the backgrounds of *fwa* introgressed into *aipp3-1*, *phd1-2*, *mom1-3* and *mom2-1*; Flowering time of MOM2-ZF T1 plants in the backgrounds of *fwa* introgressed into *aipp3-1*, *phd1-2*, *mom1-3* and *pial1/2*. **c,** Flowering time of *fwa* introgressed into *mom1-3*, *pial1/2* and *aipp3* plants, with Col-0 and *fwa* plants as controls. **d**, Flowering time of MOM2-ZF, PIAL1-ZF and PHD1-ZF T1 plants in the background of *fwa* introgressed into *mom6-3*; Flowering time of MORC6-ZF T1 plants in the backgrounds of *fwa* introgressed into *mom1-3*, *mom2-1*, *pial1/2*, *phd1-2* and *aipp3-1*. The numbers of independent plants (*n*) scored for each population and detailed statistics of flowering time comparison between different populations are listed in Supplementary Data 5. Source data are provided as a Source Data file.

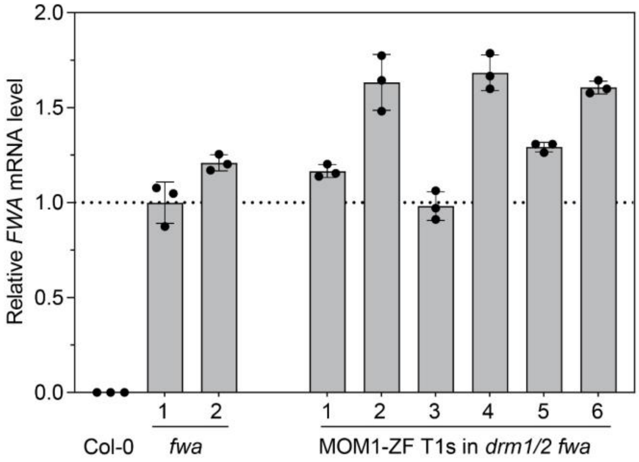

Supplementary Figure 5. *FWA* expression level of MOM1-ZF T1 plants in *drm1/2 fwa* background. Relative mRNA level of the *FWA* gene measured by qRT-PCR in the leaves of six MOM1-ZF T1 plants in *drm1/2 fwa* background which had earliest flowering time within the population (23-25 true leaves) is plotted. Bar plots and error bars indicate the mean and standard error of three technical replicates, respectively, with individual technical replicates shown as dots. Source data are provided as a Source Data file.

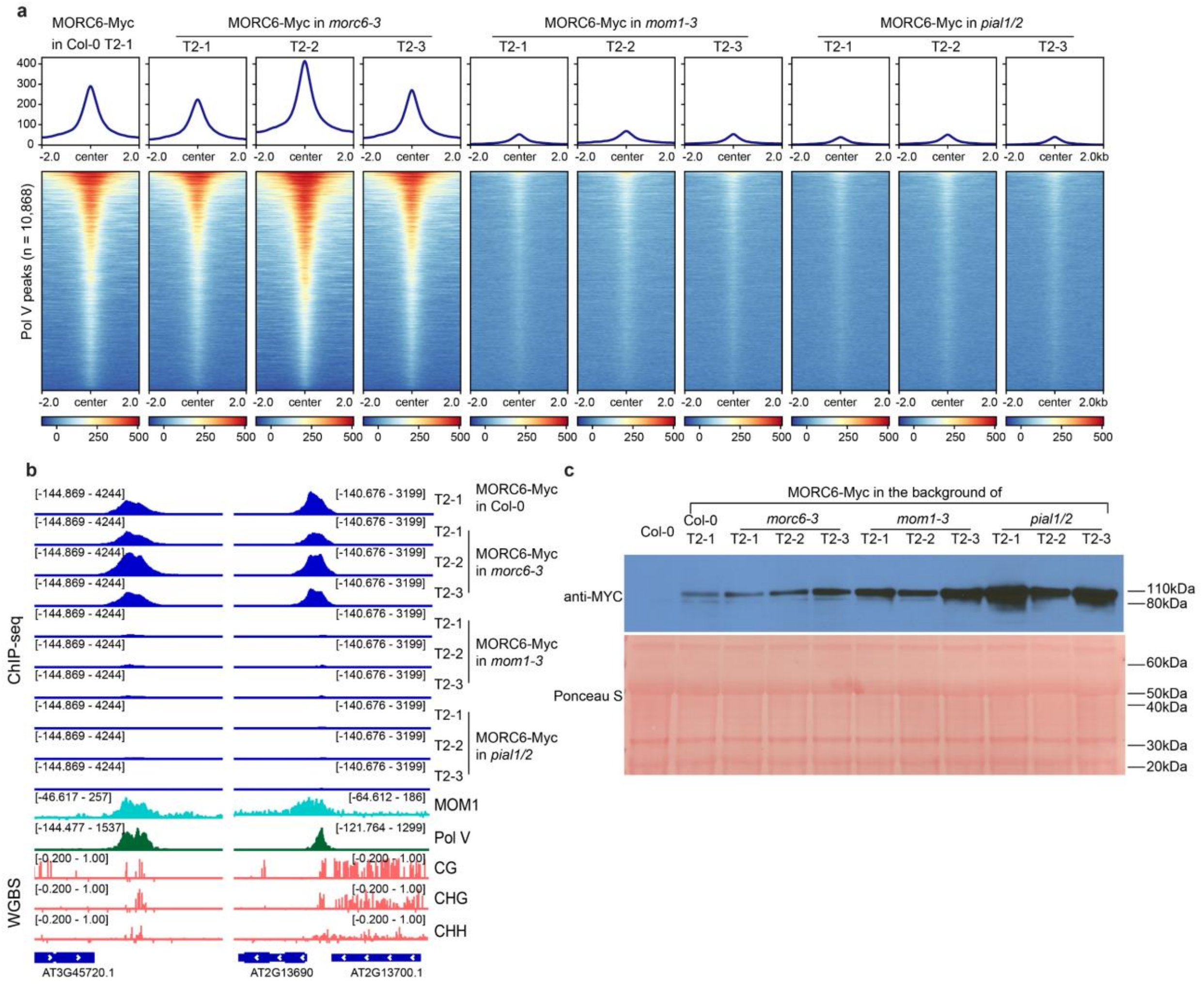

Supplementary Figure 6. Comparison of MORC6 ChIP-seq signal in Col-0, *morc6-3, mom1-3* and *pial1/2* backgrounds. a, Metaplots and heatmaps representing ChIP-seq signals of MORC6-Myc in the backgrounds of Col-0, *morc6-3* mutant, *mom1-3* mutant and *pial1/2* double mutant over Pol V peaks (n=10,868). ChIP-seq signal of control samples were subtracted for plotting. **b**, Screenshots of ChIP-seq signals of MORC6-Myc in the backgrounds of Col-0, *morc6-3* mutant, *mom1-3* mutant and *pial1/2* double mutant, as well as ChIP-seq signal of MOM1-Myc and Pol V, with control ChIP-seq signal subtracted, and CG, CHG, and CHH DNA methylation level by WGBS over representative RdDM sites. **c**, MORC6-Myc protein level of the transgenic lines used for ChIP-seq was compared by western blot. For each transgenic line, leaf discs of the same size were taken from eight hygromycin resistant T2 plants and were pooled for protein extraction. As loading control, Ponceau S staining of the same transferred membrane is shown. This experiment was repeated twice independently with similar results. Source data are provided as a Source Data file.

a

Comparison of flowering time as counted by leaf number of *FWA* transgene T1 plant populations in the following background:

background	Leaf No. Mean[1]	n	Mean Difference[2]	Adjusted P Value	P value summary
Col-0	15.91	127	0	NA	NA
nrpe1-11	33.81	16	-17.9	<0.0001	****
mom1-3	27.55	31	-11.64	<0.0001	****
pial1/2	31.98	59	-16.07	<0.0001	****
mom2-2	18.36	80	-2.449	0.1055	ns
aipp3-1	13.25	104	2.663	0.0358	*
phd1-2	16.85	73	-0.9359	0.9289	ns

[1] Leaf No. Mean = mean of (rosette + cauline leaf number)

[2] Mean difference = (mean leaf number in Col-0) - (mean leaf number in other background)

b

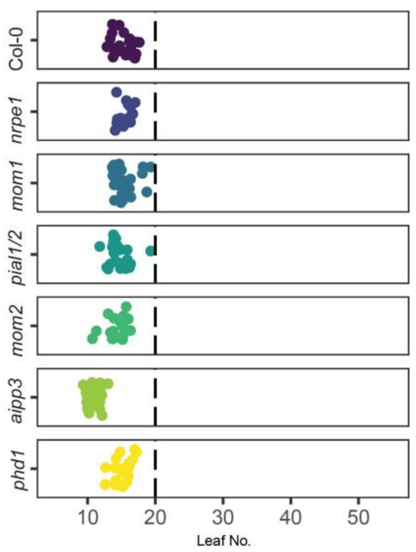

Supplementary Figure 7. Flowering time of *FWA* transgene T1 plants in MOM1 complex component mutant backgrounds. a, Comparison of the flowering time of T1 plant populations with *FWA* transgenes in the Col-0, *nrpe1-11* and MOM1 complex component mutant backgrounds. One-way ANOVA followed by Dunnett's multiple comparison tests were used for statistical analysis. Exact adjusted *P*-values for the following comparisons are: *nrpe1-11* vs Col-0, *P* = 0.00e+0; *mom1-3* vs Col-0, *P* = 7.84e-14; *pial1/2* vs Col-0, *P* = 0.00e+0. **b**, Flowering time of Col-0, *nrpe1-11, mom1-3, pial1/2, mom2-2, aipp3-1* and *phd1-2* plants. Source data are provided as a Source Data file.

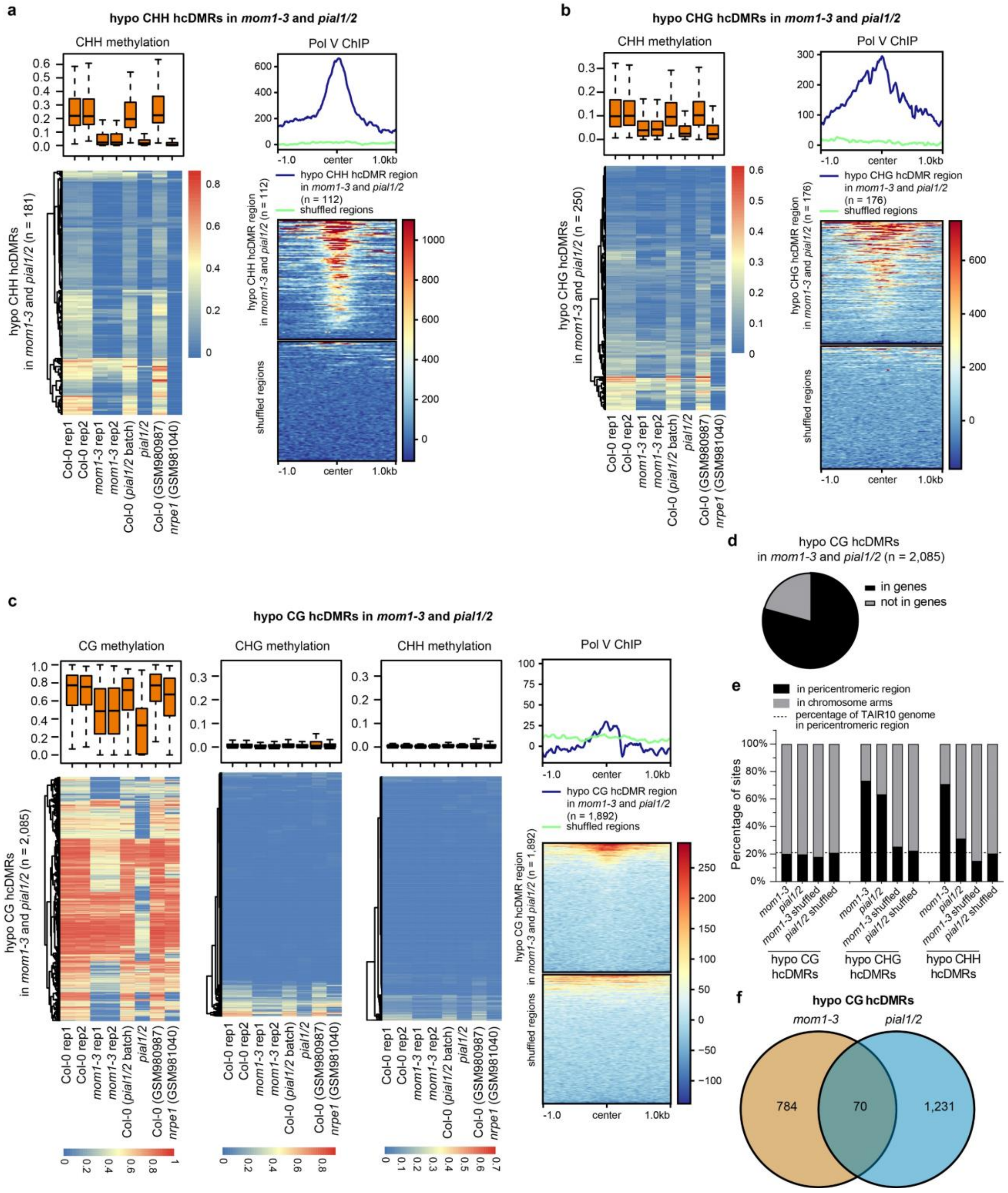

Supplementary Figure 8. Characterization of hypo CHH, hypo CHG and hypo CG hcDMRs in *mom1-3* and *pial1/2* mutants. a and **b**, left panel: Boxplot and heatmap showing DNA methylation level (in CHH context) of *mom1-3, pial1/2* and *nrpe1* mutants and Col-0 controls over pooled hypo CHH hcDMRs (**a**) and hypo CHG hcDMRs (**b**) detected in *mom1-3* and *pial1/2* mutants. Right panel: Metaplots and heatmaps representing Pol V ChIP-seq signal with control ChIP-seq signal subtracted over the regions of hypo CHH hcDMRs (**a**) and hypo CHG hcDMRs (**b**) detected in *mom1-3* and *pial1/2* mutants. Close-by hypo hcDMRs (less than 500 bp apart) were merged for plotting to avoid duplicate representation of the same region. **c**, left three panels: DNA methylation level (in CG, CHG and CHH contexts) of *mom1-3*, *pial1/2* and *nrpe1* mutants and Col-0

controls over pooled hypo CG hcDMRs detected in *mom1-3* and *pial1/2* mutants. Right panel: Metaplots and heatmaps representing Pol V ChIP-seq signal with control ChIP-seq signal subtracted over the regions of hypo CG hcDMRs detected in *mom1-3* and *pial1/2* mutants. Close-by hypo hcDMRs (less than 500 bp apart) were merged for plotting to avoid duplicate representation of the same region. **d**, Pie chart showing the localization of hypo CG hcDMRs detected in *mom1-3* and *pial1/2* mutants (pooled) relative to genes. **e,** Percentage of hypo CHH, CHG, CG hcDMRs detected in *mom1-3* and *pial1/2* mutants located in pericentromeric region and in chromosome arms. **f**, Venn diagram showing the overlapping of hypo CG hcDMRs detected in *mom1-3* and *pial1/2* mutants. In box plots of **a, b** and **c**, center line represents the median; box limits represent the 25th and 75th percentiles; whiskers represent the minimum and the maximum. Source data are provided as a Source Data file.

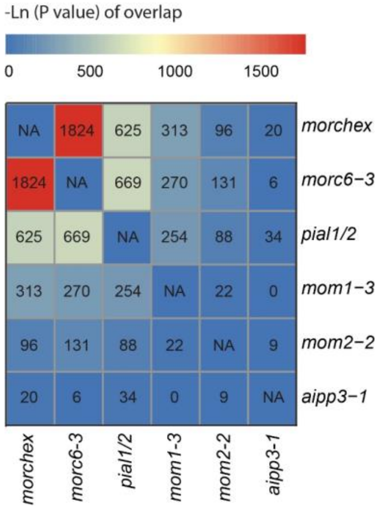

Supplementary Figure 9. Statistical analysis of the overlapping of hypo CHH hcDMRs. Heatmap of the negative natural log of *P*-value, as calculated by using the hypergeometric distribution by homer mergePeaks, for the overlapping of hypo CHH hcDMRs among *aipp3-1*, *mom2-2*, *mom1-3*, *pial1/2*, *morc6-3* and *morchex* mutants over *morchex* mutant hypo CHH hcDMRs (n=520). Source data are provided as a Source Data file.

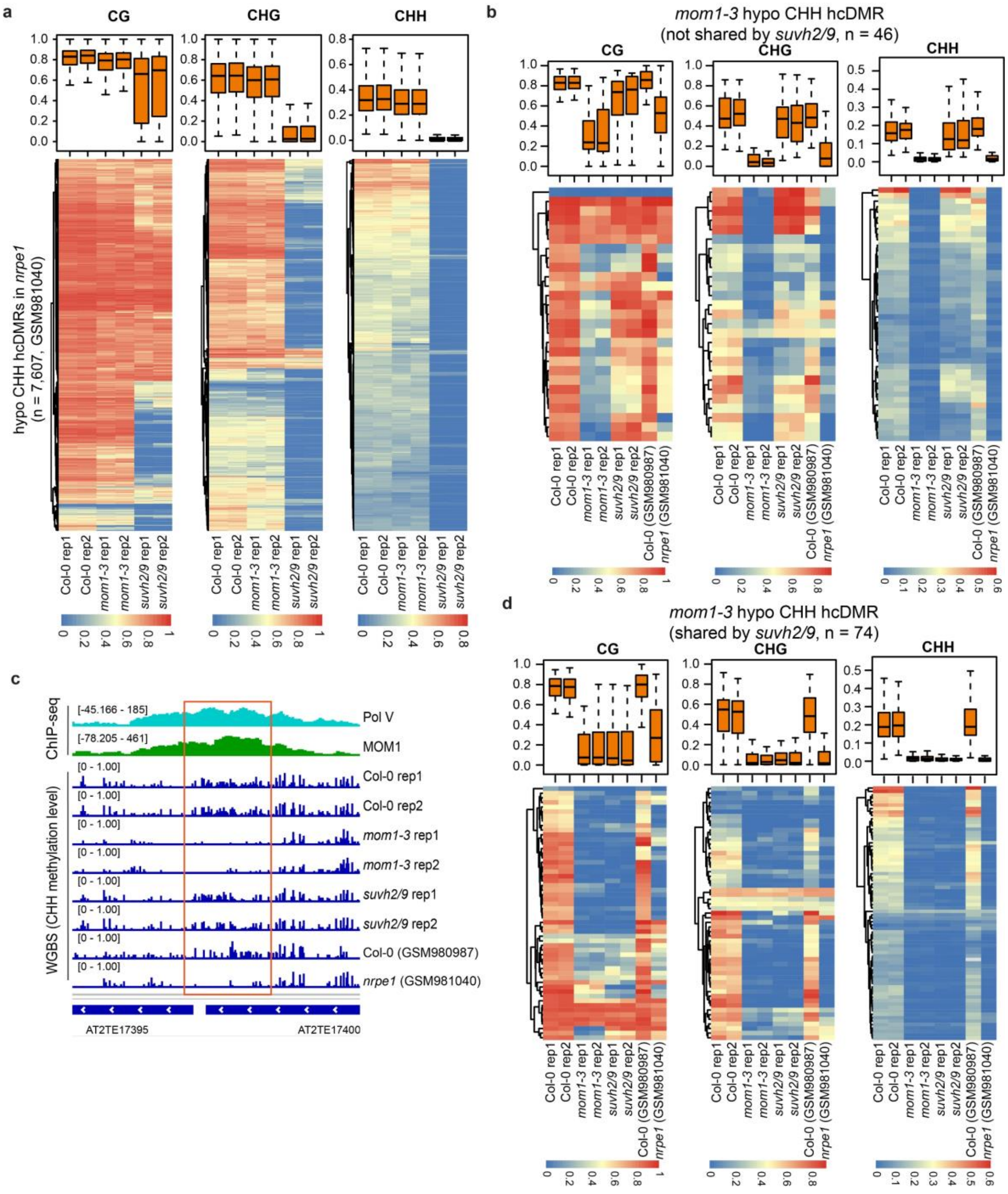

Supplementary Figure 10. MOM1 can trigger RdDM without SUVH2/9 at a small number of endogenous sites. a, Boxplots and heatmaps of DNA methylation level (in CG, CHG and CHH contexts) of *mom1-3, suvh2/9* mutants and Col-0 control over hypo CHH hcDMRs in *nrpe1* mutant (GSM981040). **b**, Boxplots and heatmaps of DNA methylation level (in CG, CHG and CHH contexts) of *mom1-3, suvh2/9, nrpe1* mutants and corresponding Col-0 controls over the hypo CHH hcDMRs which are detected in *mom1-3* mutant, but not in *suvh2/9* double mutant. **c,** Screenshots of Pol V and MOM1-Myc ChIP-seq signals with control ChIP-seq signal subtracted, as well as CHH DNA methylation level by WGBS of *mom1-3, suvh2/9, nrpe1* mutants and corresponding Col-0 controls over an example region as described in **b**. The orange rectangle indicates the region where CHH DNA methylation is dependent on MOM1 but not on SUVH2/9. **d,** Boxplots and heatmaps of DNA methylation level (in CG, CHG and CHH contexts) of *mom1-3, suvh2/9, nrpe1* mutants and corresponding Col-0 controls over the hypo CHH hcDMRs which are detected in both *mom1-3* and *suvh2/9* mutants. In box plots of **a, b** and **d**, center line represents the median; box limits represent the 25th and 75th percentiles; whiskers represent the minimum and the maximum. Source data are provided as a Source Data file.

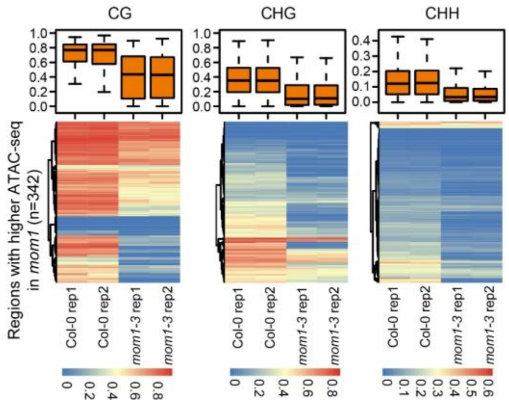

Supplementary Figure 11. DNA methylation level is decreased over regions with higher ATAC-seq signal in *mom1-3*. Boxplots and heatmaps of DNA methylation level (in CG, CHG and CHH contexts) in Col-0 and *mom1-3* mutant over the regions with higher ATAC-seq signal in *mom1-3* mutant. In box plots, center line represents the median; box limits represent the 25th and 75th percentiles; whiskers represent the minimum and the maximum. Source data are provided as a Source Data file.

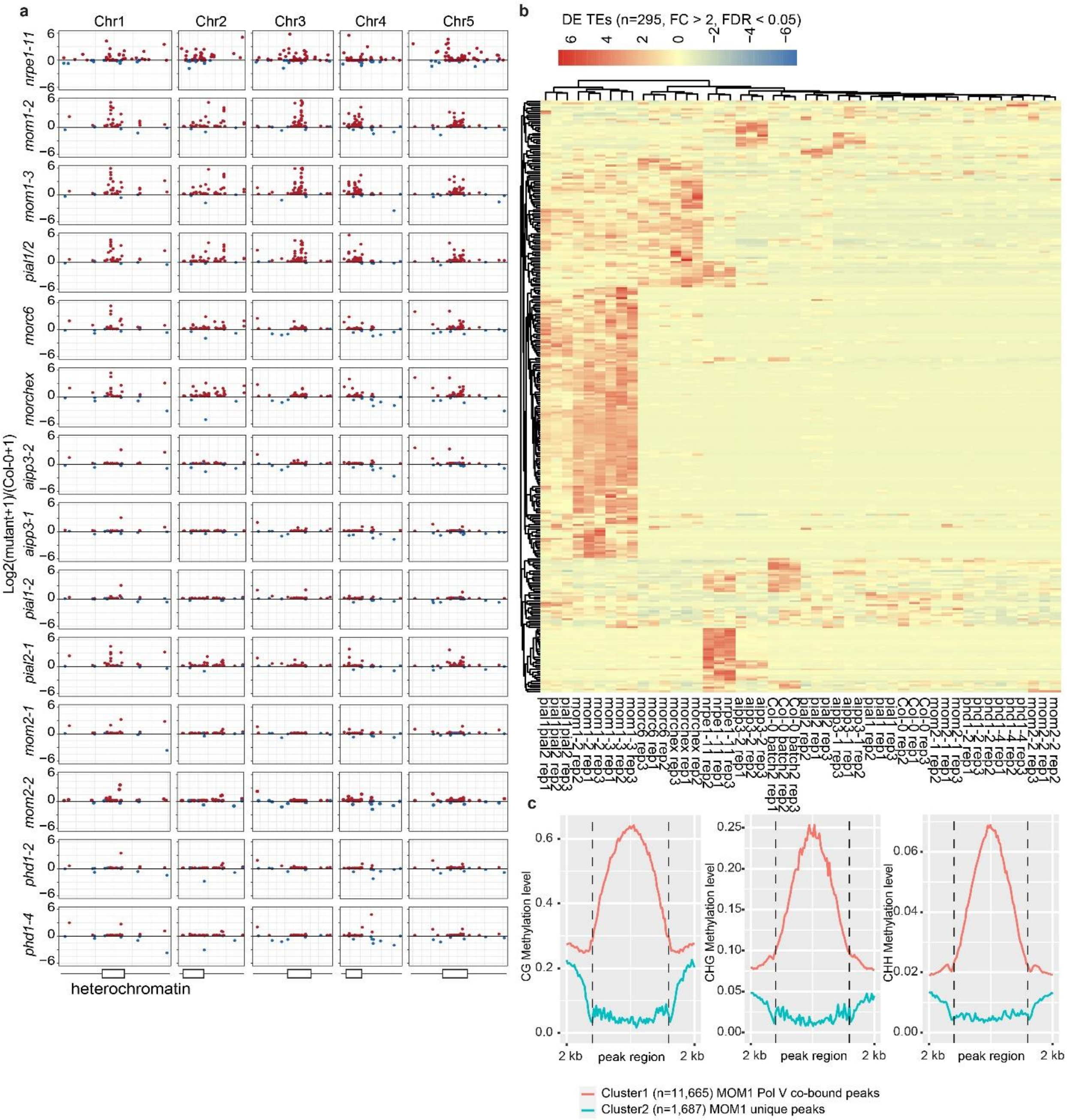

Supplementary Figure 12. RNA-seq analysis of the mutants of MOM1 complex components. **a**, Dotplots showing the differentially expressed TEs (compared to Col-0 control) over the five *Arabidopsis* chromosomes in the *nrpe1-11, mom1-2, mom1-3, pial1/2, morc6-3, morchex, aipp3-2, aipp3-1, pial1-2, pial2-1, mom2-1, mom2-2, phd1-2* and *phd1-4* mutant backgrounds. Red and blue dots indicate upregulated and down regulated TEs in mutants compared to Col-0 control, respectively. The positions of pericentromeric heterochromatin regions of each chromosome are annotated at the bottom of each plot. **b**, Heatmap showing the expression level of differentially expressed TEs (DE TEs, n=295) in three replicates of *mom1-2, mom1-3, pial1/2, morc6-3, morchex, aipp3-1, aipp3-2, pial1-2, pial2-1, mom2-1, mom2-2, phd1-2* and *phd1-4* mutant plants versus Col-0 plants. Expression level of these TEs in *nrpe1-11* mutant and corresponding Col-0 control plants are also plotted for comparison. **c**, Col-0 DNA methylation level (in CG, CHG and CHH contexts, data from GSM1375966) plotted over Cluster 1 and Cluster 2 ChIP-seq peaks of MOM1 and Pol V. Source data are provided as a Source Data file.
