# Supplementary material for: The MOM1 complex recruits the RdDM machinery via MORC6 to establish de novo DNA methylation
Source: Nat Commun. 2023 Jul 12;14:4135. doi: 10.1038/s41467-023-39751-4 (PMC10338684; doi:10.1038/s41467-023-39751-4)
Supplement: Supplementary file 3 — Description of Additional Supplementary Files [file 41467_2023_39751_MOESM3_ESM.pdf]

Description of Additional Supplementary Files

File Name: Supplementary Data 1

Description: IP-MS data.

File Name: Supplementary Data 2

Description: MOM1 crosslinking IP-MS data.

File Name: Supplementary Data 3

Description: Hypo hcDMRs in the mutants of mom1 complex.

File Name: Supplementary Data 4

Description: Primers used in this study.

File Name: Supplementary Data 5

Description: Flowering time statistics.
