# Supplementary material for: The MOM1 complex recruits the RdDM machinery via MORC6 to establish de novo DNA methylation
Source: Nat Commun. 2023 Jul 12;14:4135. doi: 10.1038/s41467-023-39751-4 (PMC10338684; doi:10.1038/s41467-023-39751-4)
Supplement: Supplementary file 9 — Reporting Summary [file 41467_2023_39751_MOESM9_ESM.pdf]

nature portfolio

Corresponding author(s): Steve Jacobsen
Last updated by author(s): Jun 16, 2023

Reporting Summary

Nature Portfolio wishes to improve the reproducibility of the work that we publish. This form provides structure for consistency and transparency in reporting. For further information on Nature Portfolio policies, see our Editorial Policies and the Editorial Policy Checklist.

Statistics

For all statistical analyses, confirm that the following items are present in the figure legend, table legend, main text, or Methods section.

n/a	Confirmed	
☐	☒	The exact sample size (*n*) for each experimental group/condition, given as a discrete number and unit of measurement
☒	☐	A statement on whether measurements were taken from distinct samples or whether the same sample was measured repeatedly
☐	☒	The statistical test(s) used AND whether they are one- or two-sided<br>*Only common tests should be described solely by name; describe more complex techniques in the Methods section.*
☒	☐	A description of all covariates tested
☒	☐	A description of any assumptions or corrections, such as tests of normality and adjustment for multiple comparisons
☐	☒	A full description of the statistical parameters including central tendency (e.g. means) or other basic estimates (e.g. regression coefficient) AND variation (e.g. standard deviation) or associated estimates of uncertainty (e.g. confidence intervals)
☐	☒	For null hypothesis testing, the test statistic (e.g. *F*, *t*, *r*) with confidence intervals, effect sizes, degrees of freedom and *P* value noted<br>*Give P values as exact values whenever suitable.*
☒	☐	For Bayesian analysis, information on the choice of priors and Markov chain Monte Carlo settings
☒	☐	For hierarchical and complex designs, identification of the appropriate level for tests and full reporting of outcomes
☒	☐	Estimates of effect sizes (e.g. Cohen's *d*, Pearson's *r*), indicating how they were calculated

Our web collection on statistics for biologists contains articles on many of the points above.

Software and code

Policy information about availability of computer code

Data collection	No software was used for data collection.
Data analysis	For IP-MS data analysis, MS/MS database searching was performed using MaxQuant (1.6.10.43) against newest Arabidopsis thaliana proteome database from http://www.uniprot.org. Analysis of raw data was obtained from the LC–MS runs using MaxQuant with the integrated Andromeda peptide search engine using default setting with enabled LFQ normalization. Data sets were filtered at a 1% FDR at both the PSM and protein levels. The MaxQuant peptide intensity and MS/MS counts were used for all peptide quantitation. For Fig. 1c, fold of change of MS/MS counts and P value of MOM1-FLAG lines crosslinking IP-MS compared to crosslinking IP-MS of Col-0 control were calculated by LIMMA (3.52.4).<br><br>For ChIP-seq analysis, raw reads were trimmed using trim_galore (https://www.bioinformatics.babraham.ac.uk/projects/trim_galore/) and aligned to the TAIR10 reference genome with bowtie2 (v2.4.2) allowing zero mismatch and reporting one valid alignment for each read. The Samtools (v1.15) were used to convert sam files to bam files, sort bam files and remove duplicate reads. Track files in bigWig format were generated using bamCoverage of deeptools (v3.5.1) with RPKM normalization. Peaks were called with MACS2 (v2.1.2) and peaks frequently identified in previous ChIP-seq of Col-0 plant with M2 antibody for FLAG epitope were removed from analysis. For unsupervised clustering of Pol V and MOM1 peaks (Fig. 6b), RPKM of Pol V, MOM1 and corresponding control ChIP-seqs over merged peaks of Pol V and MOM1 were calculated with custom scripts. Then, log2(PolV RPKM/control RPKM) and log2(MOM1 RPKM /control RPKM) were calculated and used for unsupervised clustering with the ConcensusClusterPlus R package (v1.60.0).<br><br>For RNAseq data analysis, the raw reads were aligned to the TAIR10 reference genome with bowtie2. Rsem-calculate-expression (v1.3.1) from RSEM with default settings was used to calculate expression levels. DEGs and DE-TEs were calculated with run_DE_analysis.pl from Trinity version 2.8.5 and log2 FC ≥ 1 and FDR < 0.05 were used as the cut off. RNA-seq track files in bigWig format were generated using

bamCoverage of deeptools (v3.1.3) with RPKM normalization.

For WGBS data analysis, raw reads were aligned to both strands of the TAIR10 reference genome using BSMAP (v.2.74), allowing up to 2 mismatches and 1 best hit. Reads with more than 3 consecutives methylated CHH sites were removed, and the methylation level was calculated with the ratio of C/(C+T). For Fig. 2d, the methylation levels at 1kb flanking regions of ZF off target sites in MOM1-ZF, MOM2-ZF, PIAL1-ZF, PIAL2-ZF and PHD1-ZF were subtracted by the methylation level of fwa and plotted with R package pheatmap (v1.0.12). For Fig. 5a, the hcDMRs (p < 0.01, > 33 supported controls) of Col-0 wild type, aipp3-1, phd1-2, mom1-2, mom2-1, pial1 pial2, morc6, and morchex mutants were called using a previous method, which were then used to generate the heat map using R package pheatmap (1.0.12) [R. Kolde, Pheatmap: pretty heatmaps]. For Fig. 5b, Overlap Enrichment was calculated by using HOMER (v4.11.1) mergePeaks to identify overlapped CHH hcDMR regions and followed by normalization with genome size and over random shuffles.

For BS-PCR-seq analysis, raw reads were aligned to both strands of the TAIR10 reference genome with BSMAP (v.2.90) allowing up to 2 mismatches and 1 best hit. After quality filtering, the methylation level of cytosines was calculated as the ratio of C/(C+T), and customized R scripts were used to plot methylation data over the FWA region 1-3.

For ATAC-seq data analysis, raw reads were adaptor-trimmed with trim_galore and mapped to the TAIR10 reference genome with Bowtie2 (v2.4.2) (-X 2000 -m 1). After removing duplicate reads and reads mapped to chloroplast and mitochondrial, ATAC-Seq open chromatin peaks of each replicate were called using MACS2 (v2.1.2) with parameters -p 0.01 --nomodel --shift -100 --extsize 200. Consensus peaks between replicates were identified with bedtools (version 2.26.0) intersect and differential accessible peaks were called with the R packge edgeR (version 3.30.0). Merged bigwig file of the two replicates were used for heatmap and metaplot.

For manuscripts utilizing custom algorithms or software that are central to the research but not yet described in published literature, software must be made available to editors and reviewers. We strongly encourage code deposition in a community repository (e.g. GitHub). See the Nature Portfolio guidelines for submitting code & software for further information.

Data

Policy information about availability of data

All manuscripts must include a data availability statement. This statement should provide the following information, where applicable:

- Accession codes, unique identifiers, or web links for publicly available datasets
- A description of any restrictions on data availability
- For clinical datasets or third party data, please ensure that the statement adheres to our policy

The high-throughput sequencing data generated in this study have been deposited in the National Center for Biotechnology information Gene Expression Omnibus database under accession code GSE221679 [https://www.ncbi.nlm.nih.gov/geo/query/acc.cgi?acc=GSE221679]. The mass spectrometry proteomics data generated in this study have been deposited in the ProteomeXchange Consortium via the PRIDE73 partner repository under accession code PXD039991 [https://proteomecentral.proteomexchange.org/cgi/GetDataset?ID=PXD039991]. Source data are provided as a Source Data file. The TAIR10 reference genome used in this study are available at The Arabidopsis Information Resource website [https://www.arabidopsis.org/download/index-auto.jsp%3Fdir%3D%252Fdownload_files%252FGenes%252FTAIR10_genome_release]. The Col-0 DNA methylation data used in this study for screenshots in Fig. 1b, Fig. 3c, Fig. 5e, Fig. 6e and Supplementary Fig. 6b are available in the National Center for Biotechnology information Gene Expression Omnibus database under accession code GSE124746 [https://www.ncbi.nlm.nih.gov/geo/query/acc.cgi?acc=GSE124746]. The DNA methylation data of the nrpe1 mutant and corresponding Col-0 control plants used in this study for Supplementary Fig. 8a-c and Supplementary Fig. 10b-d are available in the National Center for Biotechnology information Gene Expression Omnibus database under accession code GSE39901 [https://www.ncbi.nlm.nih.gov/geo/query/acc.cgi?acc=GSE39901]. The Col-0 DNA methylation data used in this study for Supplementary Fig. 12c are available in the National Center for Biotechnology information Gene Expression Omnibus database under accession code GSE54677 [https://www.ncbi.nlm.nih.gov/geo/query/acc.cgi?acc=GSE54677]. The Col-0 DNA methylation data used in this study for Supplementary Fig. 3a are available in the National Center for Biotechnology information Gene Expression Omnibus database under accession code GSE80302 [https://www.ncbi.nlm.nih.gov/geo/query/acc.cgi?acc=GSE80302].

Human research participants

Policy information about studies involving human research participants and Sex and Gender in Research.

Reporting on sex and gender	Our study doesn't involve human research participants.
Population characteristics	Our study doesn't involve human research participants.
Recruitment	Our study doesn't involve human research participants.
Ethics oversight	Our study doesn't involve human research participants.

Note that full information on the approval of the study protocol must also be provided in the manuscript.

Field-specific reporting

Please select the one below that is the best fit for your research. If you are not sure, read the appropriate sections before making your selection.

[x] Life sciences [ ] Behavioural & social sciences [ ] Ecological, evolutionary & environmental sciences

For a reference copy of the document with all sections, see nature.com/documents/nr-reporting-summary-flat.pdf

Life sciences study design

All studies must disclose on these points even when the disclosure is negative.

Sample size	No sample size calculation was performed. The applied sample sized for RNA-seq, WGBS, ChIP-seq, BS-PCR, etc., were selected according to public standards in the field.
Data exclusions	No data exclusion in the study.
Replication	Two replicates for ATAC-seq; Two or three replicates for ChIP-seq as indicated; Three replicates for RNA-seq; One or two replicates for WGBS as indicated; One replicate for BS-PCR-seq; Two or three replicate for IP-MS and crosslinking IP-MS as indicated; For IP-MS of MOM1-FLAG in aipp3, mom2 and aipp3mom2 mutant backgrounds, only one replicate was performed. Replicates were performed independently, with all attempts at replication successful and produced highly reproducible results.
Randomization	For all experiments, treatment and control samples were grown side by side. For flowering time analysis, random young seedlings or stratified seeds were planted on soil with fixed distance.
Blinding	No blinding used because it was largely not relevant to our study. All data were collected based on the genotype of plants, while blinding the samples during the experiments will increase the risk of mislabeling and wrong results.

Reporting for specific materials, systems and methods

We require information from authors about some types of materials, experimental systems and methods used in many studies. Here, indicate whether each material, system or method listed is relevant to your study. If you are not sure if a list item applies to your research, read the appropriate section before selecting a response.

Materials & experimental systems

n/a	Involved in the study	
☐	☒	Antibodies
☒	☐	Eukaryotic cell lines
☒	☐	Palaeontology and archaeology
☒	☐	Animals and other organisms
☒	☐	Clinical data
☒	☐	Dual use research of concern

Methods

n/a	Involved in the study	
☐	☒	ChIP-seq
☒	☐	Flow cytometry
☒	☐	MRI-based neuroimaging

Antibodies

Antibodies used	Antibody for FLAG epitope (for ChIP-seq): M2 antibody, Sigma F1804, 10 ul per ChIP added at a final dilution of 1:400<br>Antibody for MYC epitope (for ChIP-seq): Cell Signaling, 71D10, 20 ul per ChIP added at a final dilution of 1:200<br>HRP conjugated antibody for FLAG epitope (for western blot): Sigma-Aldrich ANTI-FLAG M2-peroxidase A8592, 1:7500 dilution<br>HRP conjugated antibody for MYC epitope (for western blot): Santa Cruz Biotechnology Anti-Myc/c-Myc antibody 9E10 HRP (sc-40 HRP), 1:3000 dilution
Validation	Anti-FLAG M2 antibody Sigma F1804: https://www.sigmaaldrich.com/US/en/product/sigma/f1804<br>Anti-MYC cell signaling 71D10 antibody: https://www.cellsignal.com/products/primary-antibodies/myc-tag-71d10-rabbit-mab/2278<br>HRP conjugated antibody for FLAG epitope Sigma A8592: https://www.sigmaaldrich.com/US/en/product/sigma/a8592<br>HRP conjugated antibody for MYC epitope Santa Cruz Biotechnology sc-40 HRP: https://www.scbt.com/p/c-myc-antibody-9e10?gclid=CjwKCAjwvJyjBhApEiwAWz2nLQpNcYGsOfC7x6jRDD1GtD1Y8eousO7TM84Gg9FKaaHq8gTwQEtgyhoCSMAQAvD_BwE

ChIP-seq

Data deposition

☒ Confirm that both raw and final processed data have been deposited in a public database such as GEO.

☒ Confirm that you have deposited or provided access to graph files (e.g. BED files) for the called peaks.

Data access links<br>*May remain private before publication.*	All high-throughput sequencing data generated in this study are accessible at the National Center for Biotechnology information Gene Expression Omnibus via series accession GSE221679. (also weblink here https://www.ncbi.nlm.nih.gov/geo/query/acc.cgi?acc=GSE221679).
Files in database submission	ATACseq_WT_rep1.rmdup.bw<br>ATACseq_WT_rep1_R1.fastq.gz<br>ATACseq_WT_rep1_R2.fastq.gz<br>ATACseq_WT_rep2.rmdup.bw

ATACseq_WT_rep2_R1.fastq.gz
ATACseq_WT_rep2_R2.fastq.gz
ATACseq_mom1_3_rep1.rmdup.bw
ATACseq_mom1_3_rep1_R1.fastq.gz
ATACseq_mom1_3_rep1_R2.fastq.gz
ATACseq_mom1_3_rep2.rmdup.bw
ATACseq_mom1_3_rep2_R1.fastq.gz
ATACseq_mom1_3_rep2_R2.fastq.gz
BSPCR_AIPP3_ZF_line1_with_transgene_R1_001.fastq.gz
BSPCR_AIPP3_ZF_line1_with_transgene_R2_001.fastq.gz
BSPCR_AIPP3_ZF_line1_with_transgene_methratio_type.txt
BSPCR_AIPP3_ZF_line1_withwout_transgene_R1_001.fastq.gz
BSPCR_AIPP3_ZF_line1_withwout_transgene_R2_001.fastq.gz
BSPCR_AIPP3_ZF_line1_withwout_transgene_methratio_type.txt
BSPCR_AIPP3_ZF_line2_with_transgene_R1_001.fastq.gz
BSPCR_AIPP3_ZF_line2_with_transgene_R2_001.fastq.gz
BSPCR_AIPP3_ZF_line2_with_transgene_methratio_type.txt
BSPCR_AIPP3_ZF_line2_withwout_transgene_R1_001.fastq.gz
BSPCR_AIPP3_ZF_line2_withwout_transgene_R2_001.fastq.gz
BSPCR_AIPP3_ZF_line2_withwout_transgene_methratio_type.txt
BSPCR_Col0_control_R1_001.fastq.gz
BSPCR_Col0_control_R2_001.fastq.gz
BSPCR_Col0_control_methratio_type.txt
BSPCR_MOM1_ZF_with_transgene_R1_001.fastq.gz
BSPCR_MOM1_ZF_with_transgene_R2_001.fastq.gz
BSPCR_MOM1_ZF_with_transgene_methratio_type.txt
BSPCR_MOM1_ZF_without_transgene_R1_001.fastq.gz
BSPCR_MOM1_ZF_without_transgene_R2_001.fastq.gz
BSPCR_MOM1_ZF_without_transgene_methratio_type.txt
BSPCR_MOM2_ZF_with_transgene_R1_001.fastq.gz
BSPCR_MOM2_ZF_with_transgene_R2_001.fastq.gz
BSPCR_MOM2_ZF_with_transgene_methratio_type.txt
BSPCR_MOM2_ZF_without_transgene_R1_001.fastq.gz
BSPCR_MOM2_ZF_without_transgene_R2_001.fastq.gz
BSPCR_MOM2_ZF_without_transgene_methratio_type.txt
BSPCR_PHD1_ZF_with_transgene.fastq.gz
BSPCR_PHD1_ZF_with_transgene_methratio_type.txt
BSPCR_PHD1_ZF_without_transgene.fastq.gz
BSPCR_PHD1_ZF_without_transgene_methratio_type.txt
BSPCR_PIAL1_ZF_with_transgene_R1_001.fastq.gz
BSPCR_PIAL1_ZF_with_transgene_R2_001.fastq.gz
BSPCR_PIAL1_ZF_with_transgene_methratio_type.txt
BSPCR_PIAL1_ZF_without_transgene_R1_001.fastq.gz
BSPCR_PIAL1_ZF_without_transgene_R2_001.fastq.gz
BSPCR_PIAL1_ZF_without_transgene_methratio_type.txt
BSPCR_PIAL2_ZF_with_transgene_R1_001.fastq.gz
BSPCR_PIAL2_ZF_with_transgene_R2_001.fastq.gz
BSPCR_PIAL2_ZF_with_transgene_methratio_type.txt
BSPCR_PIAL2_ZF_without_transgene_R1_001.fastq.gz
BSPCR_PIAL2_ZF_without_transgene_R2_001.fastq.gz
BSPCR_PIAL2_ZF_without_transgene_methratio_type.txt
BSPCR_fwa_control_R1_001.fastq.gz
BSPCR_fwa_control_R2_001.fastq.gz
BSPCR_fwa_control_methratio_type.txt
BSPCR_miniMOM1_ZF_with_transgene_R1_001.fastq.gz
BSPCR_miniMOM1_ZF_with_transgene_R2_001.fastq.gz
BSPCR_miniMOM1_ZF_with_transgene_methratio_type.txt
BSPCR_miniMOM1_ZF_without_transgene_R1_001.fastq.gz
BSPCR_miniMOM1_ZF_without_transgene_R2_001.fastq.gz
BSPCR_miniMOM1_ZF_without_transgene_methratio_type.txt
ChIP_AIPP3flag_rep1.bw
ChIP_AIPP3flag_rep1.fastq.gz
ChIP_AIPP3flag_rep1.narrowPeak
ChIP_AIPP3flag_rep2.bw
ChIP_AIPP3flag_rep2.fastq.gz
ChIP_AIPP3flag_rep2.narrowPeak
ChIP_MOM1myc.bw
ChIP_MOM1myc.narrowPeak
ChIP_MOM1myc_R1_001.fastq.gz

ChIP_MOM1myc_R2_001.fastq.gz
ChIP_MOM1myc_rep2.bw
ChIP_MOM1myc_rep2.narrowPeak
ChIP_MOM1myc_rep2_R1_001.fastq.gz
ChIP_MOM1myc_rep2_R2_001.fastq.gz
ChIP_MORC6myc.bw
ChIP_MORC6myc.narrowPeak
ChIP_MORC6myc_R1_001.fastq.gz
ChIP_MORC6myc_R2_001.fastq.gz
ChIP_MORC6myc_rep2.bw
ChIP_MORC6myc_rep2.narrowPeak
ChIP_MORC6myc_rep2_R1_001.fastq.gz
ChIP_MORC6myc_rep2_R2_001.fastq.gz
ChIP_PHD1flag.bw
ChIP_PHD1flag.narrowPeak
ChIP_PHD1flag_R1_001.fastq.gz
ChIP_PHD1flag_R2_001.fastq.gz
ChIP_PHD1flag_rep2.bw
ChIP_PHD1flag_rep2.fastq.gz
ChIP_PHD1flag_rep2.narrowPeak
ChIP_PHD3_rep2_myc_tag.bw
ChIP_PHD3_rep2_myc_tag.narrowPeak
ChIP_PHD3_rep2_myc_tag_R1_001.fastq.gz
ChIP_PHD3_rep2_myc_tag_R2_001.fastq.gz
ChIP_PHD3flag.bw
ChIP_PHD3flag.fastq.gz
ChIP_PHD3flag.narrowPeak
ChIP_PIAL2myc.bw
ChIP_PIAL2myc.narrowPeak
ChIP_PIAL2myc_R1_001.fastq.gz
ChIP_PIAL2myc_R2_001.fastq.gz
ChIP_PIAL2myc_rep2.bw
ChIP_PIAL2myc_rep2.narrowPeak
ChIP_PIAL2myc_rep2_R1_001.fastq.gz
ChIP_PIAL2myc_rep2_R2_001.fastq.gz
ChIP_WT_control_forAIPP3flagrep1.bw
ChIP_WT_control_forAIPP3flagrep1.fastq.gz
ChIP_WT_control_forAIPP3flagrep2.bw
ChIP_WT_control_forAIPP3flagrep2.fastq.gz
ChIP_WT_control_forMOM1myc.bw
ChIP_WT_control_forMOM1myc_R1_001.fastq.gz
ChIP_WT_control_forMOM1myc_R2_001.fastq.gz
ChIP_WT_control_forMOM1mycrep2.bw
ChIP_WT_control_forMOM1mycrep2_R1_001.fastq.gz
ChIP_WT_control_forMOM1mycrep2_R2_001.fastq.gz
ChIP_WT_control_forMORC6myc.bw
ChIP_WT_control_forMORC6myc_R1_001.fastq.gz
ChIP_WT_control_forMORC6myc_R2_001.fastq.gz
ChIP_WT_control_forMORC6mycrep2.bw
ChIP_WT_control_forMORC6mycrep2_R1_001.fastq.gz
ChIP_WT_control_forMORC6mycrep2_R2_001.fastq.gz
ChIP_WT_control_forPHD1flag.bw
ChIP_WT_control_forPHD1flag_R1_001.fastq.gz
ChIP_WT_control_forPHD1flag_R2_001.fastq.gz
ChIP_WT_control_forPHD1flagrep2.bw
ChIP_WT_control_forPHD1flagrep2.fastq.gz
ChIP_WT_control_forPHD3flag.bw
ChIP_WT_control_forPHD3flag.fastq.gz
ChIP_WT_control_forPHD3rep2myctag.bw
ChIP_WT_control_forPHD3rep2myctag_R1_001.fastq.gz
ChIP_WT_control_forPHD3rep2myctag_R2_001.fastq.gz
ChIP_WT_control_forPIAL2myc.bw
ChIP_WT_control_forPIAL2myc_R1_001.fastq.gz
ChIP_WT_control_forPIAL2myc_R2_001.fastq.gz
ChIP_WT_control_forPIAL2mycrep2.bw
ChIP_WT_control_forPIAL2mycrep2_R1_001.fastq.gz
ChIP_WT_control_forPIAL2mycrep2_R2_001.fastq.gz
ChIP_Col0rep1_forMORC6ChIPcompare.bw
ChIP_Col0rep1_forMORC6ChIPcompare_R1.fastq.gz

ChIP_Col0rep1_forMORC6ChIPcompare_R2.fastq.gz
ChIP_Col0rep2_forMORC6ChIPcompare.bw
ChIP_Col0rep2_forMORC6ChIPcompare_R1.fastq.gz
ChIP_Col0rep2_forMORC6ChIPcompare_R2.fastq.gz
ChIP_Col0MORC6Myc_T2_1.bw
ChIP_Col0MORC6Myc_T2_1_R1_001.fastq.gz
ChIP_Col0MORC6Myc_T2_1_R2_001.fastq.gz
ChIP_Col0MORC6Myc_T2_1_peaks.narrowPeak
ChIP_Col0MORC6Myc_T2_2.bw
ChIP_Col0MORC6Myc_T2_2_R1_001.fastq.gz
ChIP_Col0MORC6Myc_T2_2_R2_001.fastq.gz
ChIP_Col0MORC6Myc_T2_2_peaks.narrowPeak
ChIP_mom13MORC6Myc_T2_1.bw
ChIP_mom13MORC6Myc_T2_1_R1_001.fastq.gz
ChIP_mom13MORC6Myc_T2_1_R2_001.fastq.gz
ChIP_mom13MORC6Myc_T2_1_peaks.narrowPeak
ChIP_mom13MORC6Myc_T2_2.bw
ChIP_mom13MORC6Myc_T2_2_R1.fastq.gz
ChIP_mom13MORC6Myc_T2_2_R2.fastq.gz
ChIP_mom13MORC6Myc_T2_2_peaks.narrowPeak
ChIP_mom13MORC6Myc_T2_3.bw
ChIP_mom13MORC6Myc_T2_3_R1.fastq.gz
ChIP_mom13MORC6Myc_T2_3_R2.fastq.gz
ChIP_mom13MORC6Myc_T2_3_peaks.narrowPeak
ChIP_morc6MORC6Myc_T2_1.bw
ChIP_morc6MORC6Myc_T2_1_R1.fastq.gz
ChIP_morc6MORC6Myc_T2_1_R2.fastq.gz
ChIP_morc6MORC6Myc_T2_1_peaks.narrowPeak
ChIP_morc6MORC6Myc_T2_2.bw
ChIP_morc6MORC6Myc_T2_2_R1_001.fastq.gz
ChIP_morc6MORC6Myc_T2_2_R2_001.fastq.gz
ChIP_morc6MORC6Myc_T2_2_peaks.narrowPeak
ChIP_morc6MORC6Myc_T2_3.bw
ChIP_morc6MORC6Myc_T2_3_R1.fastq.gz
ChIP_morc6MORC6Myc_T2_3_R2.fastq.gz
ChIP_morc6MORC6Myc_T2_3_peaks.narrowPeak
ChIP_pial1pial2MORC6Myc_T2_1.bw
ChIP_pial1pial2MORC6Myc_T2_1_R1_001.fastq.gz
ChIP_pial1pial2MORC6Myc_T2_1_R2_001.fastq.gz
ChIP_pial1pial2MORC6Myc_T2_1_peaks.narrowPeak
ChIP_pial1pial2MORC6Myc_T2_2.bw
ChIP_pial1pial2MORC6Myc_T2_2_R1_001.fastq.gz
ChIP_pial1pial2MORC6Myc_T2_2_R2_001.fastq.gz
ChIP_pial1pial2MORC6Myc_T2_2_peaks.narrowPeak
ChIP_pial1pial2MORC6Myc_T2_3.bw
ChIP_pial1pial2MORC6Myc_T2_3_R1_001.fastq.gz
ChIP_pial1pial2MORC6Myc_T2_3_R2_001.fastq.gz
ChIP_pial1pial2MORC6Myc_T2_3_peaks.narrowPeak
RNAseq_WT_control_fornrpe1_rep1.genes.results
RNAseq_WT_control_fornrpe1_rep1_R1_001.fastq.gz
RNAseq_WT_control_fornrpe1_rep1_R2_001.fastq.gz
RNAseq_WT_control_fornrpe1_rep2.genes.results
RNAseq_WT_control_fornrpe1_rep2_R1_001.fastq.gz
RNAseq_WT_control_fornrpe1_rep2_R2_001.fastq.gz
RNAseq_WT_control_fornrpe1_rep3.genes.results
RNAseq_WT_control_fornrpe1_rep3_R1_001.fastq.gz
RNAseq_WT_control_fornrpe1_rep3_R2_001.fastq.gz
RNAseq_WT_control_rep1.genes.results
RNAseq_WT_control_rep1_R1_001.fastq.gz
RNAseq_WT_control_rep1_R2_001.fastq.gz
RNAseq_WT_control_rep2.genes.results
RNAseq_WT_control_rep2_R1_001.fastq.gz
RNAseq_WT_control_rep2_R2_001.fastq.gz
RNAseq_WT_control_rep3.genes.results
RNAseq_WT_control_rep3_R1_001.fastq.gz
RNAseq_WT_control_rep3_R2_001.fastq.gz
RNAseq_aipp3_1_rep1.genes.results
RNAseq_aipp3_1_rep1_R1_001.fastq.gz
RNAseq_aipp3_1_rep1_R2_001.fastq.gz

RNAseq_aipp3_1_rep2.genes.results
RNAseq_aipp3_1_rep2_R1_001.fastq.gz
RNAseq_aipp3_1_rep2_R2_001.fastq.gz
RNAseq_aipp3_1_rep3.genes.results
RNAseq_aipp3_1_rep3_R1_001.fastq.gz
RNAseq_aipp3_1_rep3_R2_001.fastq.gz
RNAseq_aipp3_2_rep1.genes.results
RNAseq_aipp3_2_rep1_R1_001.fastq.gz
RNAseq_aipp3_2_rep1_R2_001.fastq.gz
RNAseq_aipp3_2_rep2.genes.results
RNAseq_aipp3_2_rep2_R1_001.fastq.gz
RNAseq_aipp3_2_rep2_R2_001.fastq.gz
RNAseq_aipp3_2_rep3.genes.results
RNAseq_aipp3_2_rep3_R1_001.fastq.gz
RNAseq_aipp3_2_rep3_R2_001.fastq.gz
RNAseq_mom1_2_rep1.genes.results
RNAseq_mom1_2_rep1_R1_001.fastq.gz
RNAseq_mom1_2_rep1_R2_001.fastq.gz
RNAseq_mom1_2_rep2.genes.results
RNAseq_mom1_2_rep2_R1_001.fastq.gz
RNAseq_mom1_2_rep2_R2_001.fastq.gz
RNAseq_mom1_2_rep3.genes.results
RNAseq_mom1_2_rep3_R1_001.fastq.gz
RNAseq_mom1_2_rep3_R2_001.fastq.gz
RNAseq_mom1_3_rep1.genes.results
RNAseq_mom1_3_rep1_R1_001.fastq.gz
RNAseq_mom1_3_rep1_R2_001.fastq.gz
RNAseq_mom1_3_rep2.genes.results
RNAseq_mom1_3_rep2_R1_001.fastq.gz
RNAseq_mom1_3_rep2_R2_001.fastq.gz
RNAseq_mom1_3_rep3.genes.results
RNAseq_mom1_3_rep3_R1_001.fastq.gz
RNAseq_mom1_3_rep3_R2_001.fastq.gz
RNAseq_mom2_1_rep1.genes.results
RNAseq_mom2_1_rep1_R1_001.fastq.gz
RNAseq_mom2_1_rep1_R2_001.fastq.gz
RNAseq_mom2_1_rep2.genes.results
RNAseq_mom2_1_rep2_R1_001.fastq.gz
RNAseq_mom2_1_rep2_R2_001.fastq.gz
RNAseq_mom2_1_rep3.genes.results
RNAseq_mom2_1_rep3_R1_001.fastq.gz
RNAseq_mom2_1_rep3_R2_001.fastq.gz
RNAseq_mom2_2_rep1.genes.results
RNAseq_mom2_2_rep1_R1_001.fastq.gz
RNAseq_mom2_2_rep1_R2_001.fastq.gz
RNAseq_mom2_2_rep2.genes.results
RNAseq_mom2_2_rep2_R1_001.fastq.gz
RNAseq_mom2_2_rep2_R2_001.fastq.gz
RNAseq_mom2_2_rep3.genes.results
RNAseq_mom2_2_rep3_R1_001.fastq.gz
RNAseq_mom2_2_rep3_R2_001.fastq.gz
RNAseq_morc6_3_rep1.genes.results
RNAseq_morc6_3_rep1_R1_001.fastq.gz
RNAseq_morc6_3_rep1_R2_001.fastq.gz
RNAseq_morc6_3_rep2.genes.results
RNAseq_morc6_3_rep2_R1_001.fastq.gz
RNAseq_morc6_3_rep2_R2_001.fastq.gz
RNAseq_morc6_3_rep3.genes.results
RNAseq_morc6_3_rep3_R1_001.fastq.gz
RNAseq_morc6_3_rep3_R2_001.fastq.gz
RNAseq_morchex_rep1.genes.results
RNAseq_morchex_rep1_R1_001.fastq.gz
RNAseq_morchex_rep1_R2_001.fastq.gz
RNAseq_morchex_rep2.genes.results
RNAseq_morchex_rep2_R1_001.fastq.gz
RNAseq_morchex_rep2_R2_001.fastq.gz
RNAseq_morchex_rep3.genes.results
RNAseq_morchex_rep3_R1_001.fastq.gz
RNAseq_morchex_rep3_R2_001.fastq.gz

RNAseq_nrpe1_11_rep1.genes.results
RNAseq_nrpe1_11_rep1_R1_001.fastq.gz
RNAseq_nrpe1_11_rep1_R2_001.fastq.gz
RNAseq_nrpe1_11_rep2.genes.results
RNAseq_nrpe1_11_rep2_R1_001.fastq.gz
RNAseq_nrpe1_11_rep2_R2_001.fastq.gz
RNAseq_nrpe1_11_rep3.genes.results
RNAseq_nrpe1_11_rep3_R1_001.fastq.gz
RNAseq_nrpe1_11_rep3_R2_001.fastq.gz
RNAseq_phd1_2_rep1.genes.results
RNAseq_phd1_2_rep1_R1_001.fastq.gz
RNAseq_phd1_2_rep1_R2_001.fastq.gz
RNAseq_phd1_2_rep2.genes.results
RNAseq_phd1_2_rep2_R1_001.fastq.gz
RNAseq_phd1_2_rep2_R2_001.fastq.gz
RNAseq_phd1_2_rep3.genes.results
RNAseq_phd1_2_rep3_R1_001.fastq.gz
RNAseq_phd1_2_rep3_R2_001.fastq.gz
RNAseq_phd1_4_rep1.genes.results
RNAseq_phd1_4_rep1_R1_001.fastq.gz
RNAseq_phd1_4_rep1_R2_001.fastq.gz
RNAseq_phd1_4_rep2.genes.results
RNAseq_phd1_4_rep2_R1_001.fastq.gz
RNAseq_phd1_4_rep2_R2_001.fastq.gz
RNAseq_phd1_4_rep3.genes.results
RNAseq_phd1_4_rep3_R1_001.fastq.gz
RNAseq_phd1_4_rep3_R2_001.fastq.gz
RNAseq_pial1_2_rep1.genes.results
RNAseq_pial1_2_rep1_R1_001.fastq.gz
RNAseq_pial1_2_rep1_R2_001.fastq.gz
RNAseq_pial1_2_rep2.genes.results
RNAseq_pial1_2_rep2_R1_001.fastq.gz
RNAseq_pial1_2_rep2_R2_001.fastq.gz
RNAseq_pial1_2_rep3.genes.results
RNAseq_pial1_2_rep3_R1_001.fastq.gz
RNAseq_pial1_2_rep3_R2_001.fastq.gz
RNAseq_pial1pial2double_rep1.genes.results
RNAseq_pial1pial2double_rep1_R1_001.fastq.gz
RNAseq_pial1pial2double_rep1_R2_001.fastq.gz
RNAseq_pial1pial2double_rep2.genes.results
RNAseq_pial1pial2double_rep2_R1_001.fastq.gz
RNAseq_pial1pial2double_rep2_R2_001.fastq.gz
RNAseq_pial1pial2double_rep3.genes.results
RNAseq_pial1pial2double_rep3_R1_001.fastq.gz
RNAseq_pial1pial2double_rep3_R2_001.fastq.gz
RNAseq_pial2_1_rep1.genes.results
RNAseq_pial2_1_rep1_R1_001.fastq.gz
RNAseq_pial2_1_rep1_R2_001.fastq.gz
RNAseq_pial2_1_rep2.genes.results
RNAseq_pial2_1_rep2_R1_001.fastq.gz
RNAseq_pial2_1_rep2_R2_001.fastq.gz
RNAseq_pial2_1_rep3.genes.results
RNAseq_pial2_1_rep3_R1_001.fastq.gz
RNAseq_pial2_1_rep3_R2_001.fastq.gz
WGBS_MOM1_ZF.fastq.gz
WGBS_MOM1_ZF_CG.bw
WGBS_MOM1_ZF_CHG.bw
WGBS_MOM1_ZF_CHH.bw
WGBS_MOM2_ZF_CG.bw
WGBS_MOM2_ZF_CHG.bw
WGBS_MOM2_ZF_CHH.bw
WGBS_MOM2_ZF_R1_001.fastq.gz
WGBS_MOM2_ZF_R2_001.fastq.gz
WGBS_PHD1_ZF.fastq.gz
WGBS_PHD1_ZF_CG.bw
WGBS_PHD1_ZF_CHG.bw
WGBS_PHD1_ZF_CHH.bw
WGBS_PIAL1_ZF_CG.bw
WGBS_PIAL1_ZF_CHG.bw

WGBS_PIAL1_ZF_CHH.bw
WGBS_PIAL1_ZF_R1_001.fastq.gz
WGBS_PIAL1_ZF_R2_001.fastq.gz
WGBS_PIAL2_ZF_CG.bw
WGBS_PIAL2_ZF_CHG.bw
WGBS_PIAL2_ZF_CHH.bw
WGBS_PIAL2_ZF_R1_001.fastq.gz
WGBS_PIAL2_ZF_R2_001.fastq.gz
WGBS_WT_control_for_aipp31mom22_CG.bw
WGBS_WT_control_for_aipp31mom22_CHG.bw
WGBS_WT_control_for_aipp31mom22_CHH.bw
WGBS_WT_control_for_aipp31mom22_R1_001.fastq.gz
WGBS_WT_control_for_aipp31mom22_R2_001.fastq.gz
WGBS_WT_control_for_phd1_3.fastq.gz
WGBS_WT_control_for_phd1_3_CG.bw
WGBS_WT_control_for_phd1_3_CHG.bw
WGBS_WT_control_for_phd1_3_CHH.bw
WGBS_aipp3_1_CG.bw
WGBS_aipp3_1_CHG.bw
WGBS_aipp3_1_CHH.bw
WGBS_aipp3_1_R1_001.fastq.gz
WGBS_aipp3_1_R2_001.fastq.gz
WGBS_fwa.fastq.gz
WGBS_fwa_CG.bw
WGBS_fwa_CHG.bw
WGBS_fwa_CHH.bw
WGBS_mom2_2_CG.bw
WGBS_mom2_2_CHG.bw
WGBS_mom2_2_CHH.bw
WGBS_mom2_2_R1_001.fastq.gz
WGBS_mom2_2_R2_001.fastq.gz
WGBS_phd1_3.fastq.gz
WGBS_phd1_3_CG.bw
WGBS_phd1_3_CHG.bw
WGBS_phd1_3_CHH.bw
WGBS_WT_control_for_mom1_3suvh2suvh9_rep1_CG.bw
WGBS_WT_control_for_mom1_3suvh2suvh9_rep1_CHG.bw
WGBS_WT_control_for_mom1_3suvh2suvh9_rep1_CHH.bw
WGBS_WT_control_for_mom1_3suvh2suvh9_rep1_R1.fastq.gz
WGBS_WT_control_for_mom1_3suvh2suvh9_rep1_R2.fastq.gz
WGBS_WT_control_for_mom1_3suvh2suvh9_rep2_CG.bw
WGBS_WT_control_for_mom1_3suvh2suvh9_rep2_CHG.bw
WGBS_WT_control_for_mom1_3suvh2suvh9_rep2_CHH.bw
WGBS_WT_control_for_mom1_3suvh2suvh9_rep2_R1.fastq.gz
WGBS_WT_control_for_mom1_3suvh2suvh9_rep2_R2.fastq.gz
WGBS_mom1_3_rep1_CG.bw
WGBS_mom1_3_rep1_CHG.bw
WGBS_mom1_3_rep1_CHH.bw
WGBS_mom1_3_rep1_R1.fastq.gz
WGBS_mom1_3_rep1_R2.fastq.gz
WGBS_mom1_3_rep2_CG.bw
WGBS_mom1_3_rep2_CHG.bw
WGBS_mom1_3_rep2_CHH.bw
WGBS_mom1_3_rep2_R1.fastq.gz
WGBS_mom1_3_rep2_R2.fastq.gz
WGBS_pial1pial2double_CG.bw
WGBS_pial1pial2double_CHG.bw
WGBS_pial1pial2double_R1.fastq.gz
WGBS_pial1pial2double_R2.fastq.gz
WGBS_suvh2suvh9double_rep1_CG.bw
WGBS_suvh2suvh9double_rep1_CHG.bw
WGBS_suvh2suvh9double_rep1_CHH.bw
WGBS_suvh2suvh9double_rep1_R1.fastq.gz
WGBS_suvh2suvh9double_rep1_R2.fastq.gz
WGBS_suvh2suvh9double_rep2_CG.bw
WGBS_suvh2suvh9double_rep2_CHG.bw
WGBS_suvh2suvh9double_rep2_CHH.bw
WGBS_suvh2suvh9double_rep2_R1.fastq.gz
WGBS_suvh2suvh9double_rep2_R2.fastq.gz

Genome browser session
(e.g. UCSC)

available at GEO

Methodology

Replicates

Two replicates for ATAC-seq; Two or three replicates for ChIP-seq; Three replicates for RNA-seq; One or two replicates for WGBS and one replicate for BS-PCR-seq.

Sequencing depth

Name Total_reads Unique_reads Reads_length Reads_type
ChIP_MOM1myc 59859152 52030347 50 PE
ChIP_MOM1myc_rep2 79756692 74082241 50 PE
ChIP_MORC6myc 53849816 48398602 50 PE
ChIP_MORC6myc_rep2 49128547 45457108 100 PE
ChIP_PHD1flag 28309839 24947216 50 PE
ChIP_PHD3_rep2_myc_tag 70835523 65483464 100 PE
ChIP_PIAL2myc 98202159 69366217 50 PE
ChIP_PIAL2myc_rep2 34902948 28395662 150 PE
ChIP_WT_control_forMOM1myc 80762965 75357037 50 PE
ChIP_WT_control_forMOM1mycrep2 66453007 63235689 50 PE
ChIP_WT_control_forMORC6myc 36713639 26210720 50 PE
ChIP_WT_control_forMORC6mycrep2 74397289 66875348 100 PE
ChIP_WT_control_forPHD1flag 30867948 27616639 50 PE
ChIP_WT_control_forPHD3rep2myctag 65162052 59394577 100 PE
ChIP_WT_control_forPIAL2myc 63580657 50800052 50 PE
ChIP_WT_control_forPIAL2mycrep2 43689278 38476739 150 PE
ChIP_AIPP3flag_rep1 36847061 35149344 100 single
ChIP_AIPP3flag_rep2 34521536 33222877 100 single
ChIP_PHD1flag_rep2 21410665 18378026 50 single
ChIP_PHD3flag 25644581 25356262 50 single
ChIP_WT_control_forAIPP3flagrep1 22908850 21698806 100 single
ChIP_WT_control_forAIPP3flagrep2 29837343 28122155 100 single
ChIP_WT_control_forPHD1flagrep2 14880957 14301852 50 single
ChIP_WT_control_forPHD3flag 25244072 24895622 50 single
ChIP_Col0rep1 46494199 45889955 33270521 100 PE
ChIP_Col0rep2 40624033 40143263 29039948 100 PE
ChIP_Col0MORC6Myc_T2_1 33801776 33485357 20525277 100 PE
ChIP_Col0MORC6Myc_T2_2 34041608 33724052 19384794 100 PE
ChIP_mom13MORC6Myc_T2_1 36555685 36231117 22968266 100 PE
ChIP_mom13MORC6Myc_T2_2 32420306 32109823 19057878 100 PE
ChIP_mom13MORC6Myc_T2_3 37340208 36975808 22383390 100 PE
ChIP_morc6MORC6Myc_T2_1 36044021 35585186 21321680 100 PE
ChIP_morc6MORC6Myc_T2_2 35672841 35369115 20615534 100 PE
ChIP_morc6MORC6Myc_T2_3 32692336 32436089 19706090 100 PE
ChIP_pial1pial2MORC6Myc_T2_1 37288577 37002967 23887064 100 PE
ChIP_pial1pial2MORC6Myc_T2_2 36633859 36367458 23434628 100 PE
ChIP_pial1pial2MORC6Myc_T2_3 33501497 33063774 20434211 100 PE

Antibodies

Anti-FLAG antibody: M2 monoclonal antibody from sigma (F1804), 10 ul per ChIP added at a final dilution of 1:400
Anti-MYC antibody: cell signaling antibody 71D10, 20 ul per ChIP added at a final dilution of 1:200

Peak calling parameters

MACS2: -f BAM -g 119146348 -q 0.01 --extsize 200

Data quality

All identified peaks in this study were called with q-value threshold as 0.01 (FDR as 1%)

Software

bowtie2 (v2.4.2)
Samtools (v1.15)
deeptools (v3.5.1)
MACS2 (v2.1.2)
bedtools (v2.30.0)
ConcensusClusterPlus R package (v1.60.0)
